# Metabolic regulation of *Escherichia coli *and its *gdhA, glnL, gltB, D *mutants under different carbon and nitrogen limitations in the continuous culture

**DOI:** 10.1186/1475-2859-9-8

**Published:** 2010-01-27

**Authors:** Rahul Kumar, Kazuyuki Shimizu

**Affiliations:** 1 Department of Bioscience and Bioinformatics, Kyushu Institute of Technology, Iizuka, Fukuoka 820-8502, Japan; 2 Institute of Advanced Bioscience, Keio University, Tsuruoka, Yamagada 997-001, Japan

## Abstract

**Background:**

It is quite important to understand how the central metabolism is regulated under nitrogen (N)- limitation as well as carbon (C)- limitation. In particular, the effect of C/N ratio on the metabolism is of practical interest for the heterologous protein production, PHB production, etc. Although the carbon and nitrogen metabolisms are interconnected and the overall mechanism is complicated, it is strongly desirable to clarify the effects of culture environment on the metabolism from the practical application point of view.

**Results:**

The effect of C/N ratio on the metabolism in *Escherichia coli *was investigated in the aerobic continuous culture at the dilution rate of 0.2 h^-1 ^based on fermentation data, transcriptional RNA level, and enzyme activity data. The glucose concentration was kept at 10 g/l, while ammonium sulfate concentration was varied from 5.94 to 0.594 g/l. The resultant C/N ratios were 1.68 (100%), 2.81(60%), 4.21(40%), 8.42(20%), and 16.84(10%), where the percentage values in brackets indicate the ratio of N- concentration as compared to the case of 5.94 g/l of ammonium sulfate. The mRNA levels of *crp *and *mlc *decreased, which caused *ptsG *transcript expression to be up-regulated as C/N ratio increased. As C/N ratio increased *cra *transcript expression decreased, which caused *ptsH, pfkA*, and *pykF *to be up-regulated. At high C/N ratio, transcriptional mRNA level of *soxR/S *increased, which may be due to the activated respiratory chain as indicated by up-regulations of such genes as *cyoA, cydB, ndh *as well as the increase in the specific CO_2 _production rate. The *rpoN *transcript expression increased with the increase in C/N ratio, which led *glnA, L, G *and *gltD *transcript expression to change in similar fashion. The *nac *transcript expression showed similar trend as *rpoN*, while *gdhA *transcript expression changed in reverse direction. The transcriptional mRNA level of *glnB*, which codes for P_II_, *glnD *and *glnK *increased as C/N ratio increases. It was shown that GS-GOGAT pathway was activated for *gdhA *mutant under N- rich condition. In the case of *glnL *mutant, GOGAT enzyme activity was reduced as compared to the wild type under N- limitation. In the case of *gltB, D *mutants, GDH and GS enzymes were utilized under both N- rich and N- limited conditions. In this case, the transcriptional mRNA level of *gdhA *and corresponding GDH enzyme activity was higher under N- limitation as compared to N- rich condition.

**Conclusion:**

The metabolic regulation of *E.coli *was clarified under both carbon (C)- limitation and nitrogen (N)- limitation based on fermentation, transcriptional mRNA level and enzyme activities. The overall regulation mechanism was proposed. The effects of knocking out N- assimilation pathway genes were also clarified.

## Background

It is quite important to understand how the culture environment affects the cell metabolism. Among the culture environments, carbon and nitrogen sources are by far important in practice. To understand the regulation of central metabolism in *E.coli*, the cell metabolism under carbon (C) and nitrogen (N) limitations has been investigated in the continuous culture [1-3]. These studies highlight the importance of critical nodal points like PEP-PYR-OAA for the carbon catabolism [4], and glutamine and glutamate synthesis for nitrogen assimilation [5]. The subsequent studies made it clear that carbon metabolism is not only controlled by carbon-derived signals, but also by the availability of nitrogen and other nutrients [6]. Therefore, several studies have been made on the regulatory interdependence of different metabolic routes. From these studies, two of the major signal transduction systems of nitrogen and carbon metabolism have been identified as P_II_, a small nitrogen regulatory protein and the phosphotransferase system (PTS). Because of the important roles in regulatory functions, P_II _and the PTS can be regarded as the central processing units of nitrogen and carbon metabolism, respectively. The P_II _protein senses α-KG and ATP, thus link the state of central carbon and energy metabolism for the control of nitrogen assimilation [6]. The glucose catabolism is modulated by the global regulators encoded by such genes as *cra, crp, cya, mlc *etc [7,8], while nitrogen assimilation is regulated by P_II_-Ntr system together with global regulators like Crp, providing a novel regulatory network between carbon and nitrogen assimilation in *E.coli *[9].

**Figure 1:**
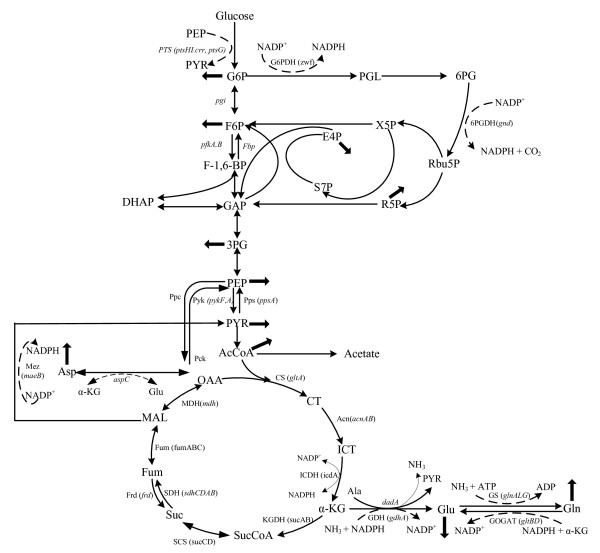
**Central metabolic pathways of *E.coli *concerned with C- metabolism and N- assimilation**.

In *E.coli*, assimilation of N-source such as ammonia/ammonium (NH_4_^+^) using α-KG results in the synthesis of glutamate and glutamine (Fig. 1). Glutamine synthetase (GS, encoded by *glnA*) catalyzes the only pathway for glutamine biosynthesis. Glutamate can be synthesized by two pathways through the combined actions of GS and glutamate synthase (GOGAT, encoded by *gltBD*) forming GS/GOGAT cycle, or by glutamate dehydrogenase (GDH, encoded by *gdhA*) [10]. The GS/GOGAT cycle has a high affinity for NH_4_^+ ^(K_m _< 0.2 mM for GS), and therefore, is dominant when nitrogen is scarce in the medium, whereas GDH has a low affinity for NH_4_^+ ^(K_m _> 1 mM), and is utilized when sufficient nitrogen is available in the medium. When extracellular NH_4_^+ ^concentration is low around 5 μM or less, ammonium enters the cell via AmtB and is converted to glutamine by GS, and UTase uridylylates both GlnK and GlnB [11]. When extracellular NH_4_^+ ^concentration is more than 50 μM, the metabolic demand for glutamine pool rises, and UTase deuridylylates GlnK and GlnB. GlnK complexes with AmtB, thereby inhibiting the transporter via AmtB, where GlnB interacts with NtrB and activates its phosphatase activity leading to dephosphorylation of NtrC, and NtrC-dependent gene expression ceases [11]. The central nitrogen intermediates such as glutamine and glutamate provide nitrogen for the synthesis of all the other N- containing components. About 88% of cellular nitrogen comes from glutamate, and the rest from glutamine [5]. The ATP required for the nitrogen assimilation using GS/GOGAT cycle under N- limiting condition accounts for 15% of the total requirement in *E.coli*. A significant amount of NADPH is also required for nitrogen assimilation [5,10]. The other pathways involved in maintaining cellular nitrogen balance under specific conditions include aspartate-oxaloacetate and alanine-pyruvate shunts [12,13].

The carbon and nitrogen metabolism are linked by energy metabolism. The glycolytic flux in *E. coli *is controlled by the demand for ATP [14]. Recently, it has been reported that the P_II _protein controls nitrogen assimilation by acting as a sensor of adenylate energy charge, which is the measure of energy available for metabolism. The signal transduction requires ATP binding to P_II_, which is synergistic with the binding of α-KG. Furthermore, α-KG serves as a cellular signal of carbon and nitrogen status, and strongly regulates P_II _functions [15]. The studies on the carbon and nitrogen pathway interdependence have so far focused on the conversion of α-KG to glutamate [16]. It is evident that the regulatory mechanism of this conversion is critical for the interdependence of carbon and nitrogen assimilation. However, little has been investigated on how gene-level regulation affects the cell metabolism under nitrogen and carbon limitations. In the present research, the effects of C/N ratio on *E.coli *metabolism based on fermentation, transcriptional mRNA levels, and enzyme activities were investigated. Such investigation is also of practical interest for the efficient production of PHB, ergosterol, ornithine, arginine, putrescine, GABA, lycopene, ε- caprolactone, etc [17-20]. Moreover, the metabolic regulation analysis can be utilized for the dynamic modeling of N- regulation [21]. In order to understand the metabolic regulation mechanism in more details, we also investigated the effects of deleting N- assimilating pathway genes such as *gdhA, glnL, gltB*, and *gltD *on the metabolism as compared to the wild type *E.coli*.

## Results

### Wild Type

**Figure 2:**
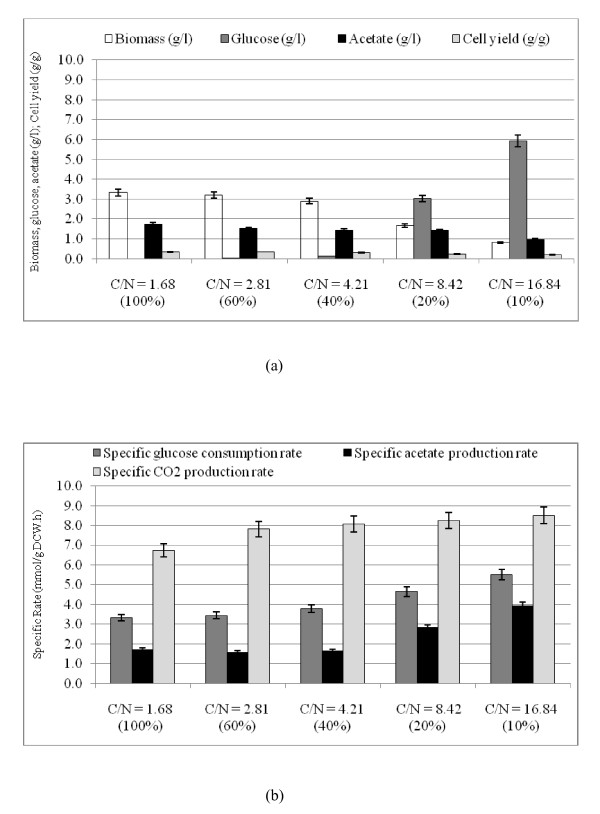
**Comparison of fermentor characteristics of the wild type *E.coli *at various C/N ratios: (a) biomass, glucose and acetate concentration (g/l), cell yield (g/g); (b) specific rates of glucose consumption, acetate and CO_2 _production (mmol/g DCW. h)**.

Fig. 2 shows the effect of C/N ratio on the fermentation characteristics, where Fig. 2a indicates that the glucose concentration increases, whereas the cell concentration decreases as C/N ratio increases. Fig. 2a also shows that the glucose concentration was very low at 100% and 60% of N concentrations (C-limitation), whereas its concentration was high at 20% and 10% of N concentrations (N-limitation). Fig. 2b shows the effect of C/N ratio on the specific rates, which indicates that the specific glucose consumption rate as well as the specific acetate and CO_2 _production rates tended to increase as C/N ratio increases. The raw fermentation data are given in Additional file 1.

**Figure 3:**
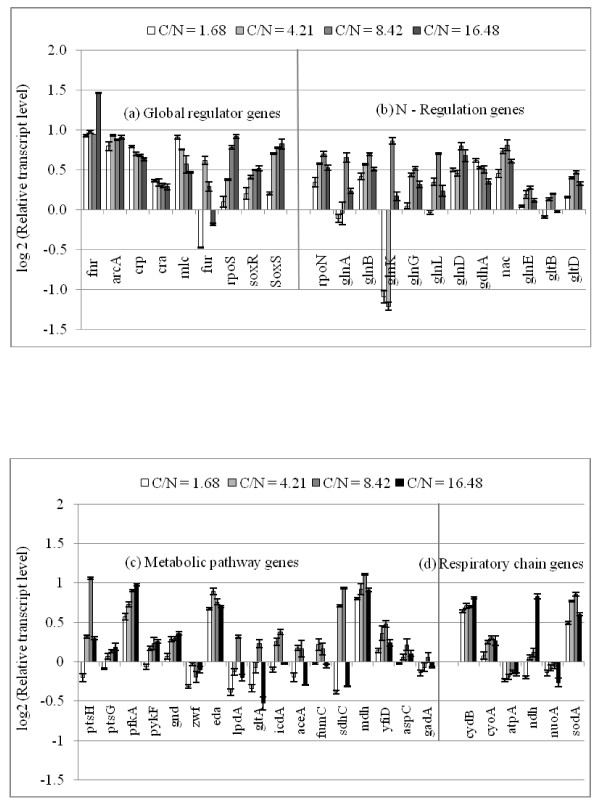
**Comparison of the transcriptional mRNA levels of the wild type *E.coli *genes cultivated at 100% (C/N = 1.68), 40% (C/N = 4.21), 20% (C/N = 8.42) and 10% (C/N = 1.68) N- concentration: (a) global regulatory, (b) N- regulatory, (c) metabolic pathway, (d) respiratory chain**.

In order to interpret the fermentation characteristics, the relative mRNA levels were measured for different C/N ratios by RT-PCR (Fig. 3). Fig 3a shows that *crp *transcript level became lower (p < 0.01) as C/N ratio increases, which corresponds to the fact that cAMP-Crp level decreases as glucose concentration increases. In accordance with the change in *crp *transcript level, *mlc *level changed in similar fashion [8]. Fig. 3a also shows that the transcript levels of such genes as *soxR/S *and *rpoS *became higher as C/N ratio increases, which may be due to oxygen stress caused by higher respiratory activity for the former [1], along with nutrient stress for the latter [22]. In relation to the up-regulation of *soxR/S*, the *sodA *transcript level increased as C/N ratio increases except at the highest C/N ratio (Fig. 3d). Fig. 3a shows quite high expression of anaerobic regulator *fnr *at the highest C/N ratio, while the transcript level of *arcA *which codes for microaerobic regulator did not change much.

The transcript level of *rpoN*, which encodes σ^54^, increased as C/N ratio increases (Fig. 3b). Fig 3b also shows that the expressions of *glnA, glnL, glnG*, and *gltD *genes changed in similar fashion as *rpoN*, indicating the activation of GS-GOGAT pathway under N-limitation. The *glnB *gene which codes for P_II _also changed in similar fashion, while *glnD *which controls the uridylylation and deuridylylation shows somewhat different but the trend seems to be similar (Fig. 3b). P_II _paralogue encoding gene, *glnK *shows very high expressions at 20% and 10% of N-limitation (Fig. 3b). The expression pattern of *nac *is similar to that of *rpoN*, whereas *gdhA *shows reverse pattern, implying that *gdhA *is repressed by Nac (Fig. 3b).

As C/N ratio increases, the transcript level of *crp *gene as well as *mlc *gene decreased, which then caused the transcript level of *pts*G gene to be increased as shown in Fig. 3c. In relation to the decrease in the transcript level of *cra*, the transcript levels of such genes as *ptsH*, *pfkA *and *pykF *increased as C/N ratio increases (Fig. 3c). These correspond to the increased specific glucose consumption rate as C/N ratio increases. Moreover, the respiratory chain genes such as *cyoA, cydB*, and *ndh *together with TCA cycle genes such as *gltA, icdA, fumC, sdhC*, and *mdh *showed increased expressions as C/N ratio increases (Fig. 3d), which corresponds to the increase in the specific CO_2 _production rate (Fig. 2b). Part of the reason why this happened may be due to the accumulation of α- KG caused by the decreased activity of GDH. Although TCA cycle genes are under control of ArcA (Additional file 2), Fig. 3a shows little change in the transcript level of *arcA *gene. Since ferric uptake regulator Fur activates some of the TCA cycle genes such as *sdh, suc*, and *fum *[23], part of the reason may be due to up-regulation of the transcript level of *fur *gene, where it is not clear at this time why the transcript level of this gene tends to decrease as C/N ratio increases (Fig. 3a).

### Mutants

**Figure 4:**
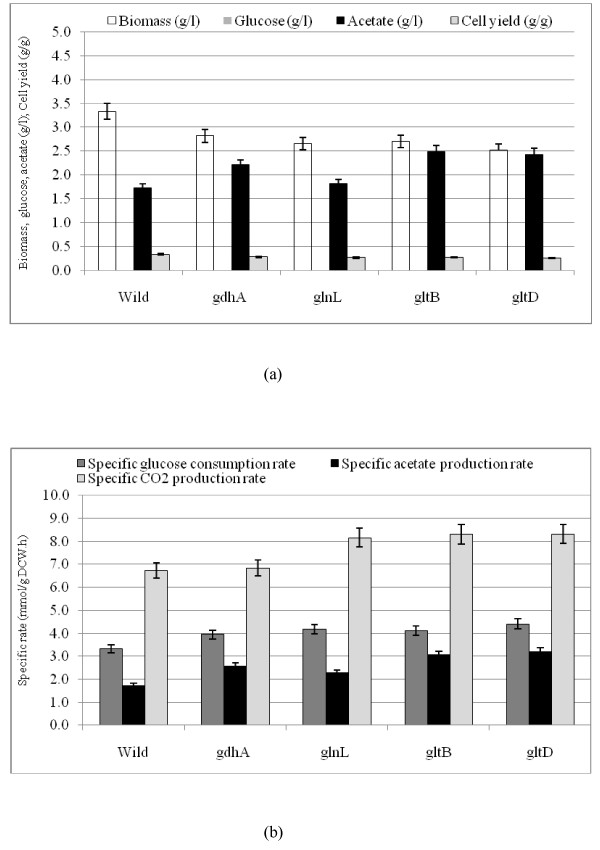
**Comparison of fermentor characteristics of the wild type *E.coli *and nitrogen assimilation related mutants, *gdhA, glnL, gltB*, and *gltD *at 100% N- concentration (C/N ratio = 1.68): (a) biomass, glucose and acetate concentration (g/l), cell yield (g/g); (b) specific rates of glucose consumption, acetate and CO_2 _production (mmol/g DCW. h)**.

**Figure 5:**
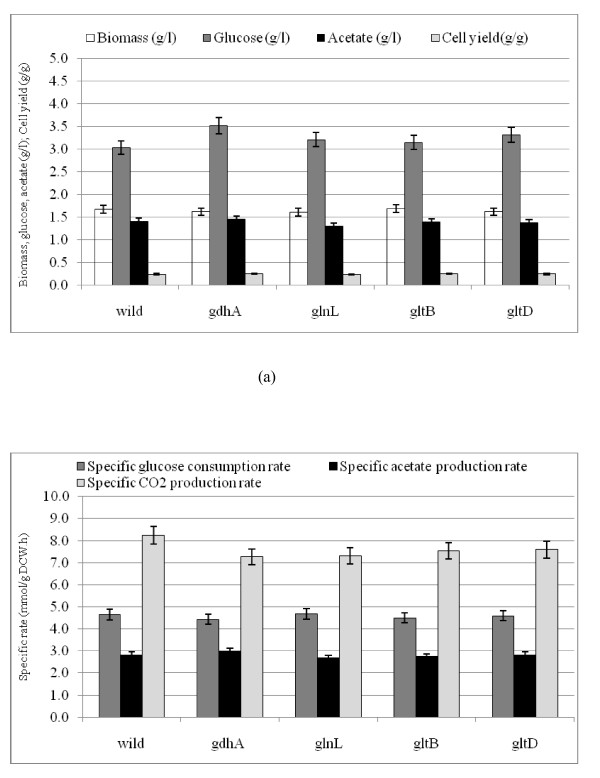
**Comparison of fermentor characteristics of the wild type *E.coli *and nitrogen assimilation related mutants, *gdhA, glnL, gltB*, and *gltD *at 20% N- concentration (C/N ratio = 8.42): (a) biomass, glucose and acetate concentration (g/l), cell yield (g/g); (b) specific rates of glucose consumption, acetate and CO_2 _production (mmol/g DCW. h)**.

Figs. 4 and 5 show the comparisons of the fermentation data for *gdhA, glnL, gltB*, and *gltD *mutants as compared to the wild type under N-rich and N- limiting conditions, where the raw data are given in Additional file 3a and 3b. Fig. 4a indicates that the cell concentrations and the cell yields of the mutants were all lower as compared to those of the wild type, where glucose was nearly completely consumed under N-rich condition. Fig. 4a also indicates that acetate concentrations increased for all the mutants as compared to the wild type. Among the mutants, acetate concentration was the lowest for *glnL *mutant followed by *gdhA, gltB *and *gltD *mutants. Fig. 4b indicates that the specific glucose consumption rate, specific acetate and CO_2 _production rates were all increased for the mutants as compared to the wild type. Fig. 5a indicates that the cell and acetate concentrations and the cell yields of the mutants were almost similar as compared to those of the wild type under N- limitation. The glucose concentration was relatively higher for the mutants as compared to the wild type (Fig. 5a), although the specific glucose consumption rates showed little changes (Fig. 5b). The former phenomenon may be caused by the lower cell concentration. Fig. 5b also indicates that the specific CO_2 _production rates for all the mutants were lower as compared to that of the wild type. Note that the specific CO_2 _production rates of *glnL, gltB, gltD *mutants as well as the wild type were higher under N-limitation as compared to those under N-rich condition, whereas the specific CO_2 _production rate of *gdhA *mutant was similar under N-limitation as compared to N-rich condition. In the following sections, the comparisons of the transcript levels between the wild type and *gdhA, glnL, gltB *and *gltD *mutants are made. Overall, the changing patterns of *gltB *and *gltD *mutants were similar, whereas *gdhA *and *glnL *mutants show somewhat different patterns.

### *gdhA *mutant

In the case where *gdhA *gene was knocked out, glutamate and glutamine were formed by GS and GOGAT pathways under N-rich condition as can be seen from the up-regulations of the transcript levels of *glnA, L, G *(p < 0.01, p < 0.01, p < 0.01, respectively), and *gltD *gene (p < 0.01) as shown in Additional file 4b. The changing patterns were similar to those of the wild type under N-limitation (Fig. 3b). Additional file 4e indicates that PTS and glycolysis genes such as *ptsH, ptsG, pfkA, pykF *genes were up-regulated (p < 0.01, p < 0.05, p < 0.01, p < 0.01, respectively) and PP pathway and ED pathway genes such as *zwf, gnd*, and *eda *genes as well as *lpdA *gene were up-regulated (p < 0.01, p < 0.01, p < 0.05, p < 0.01 respectively) for *gdhA *mutant as compared to the wild type under N-rich condition. Moreover, TCA cycle genes such as *gltA, icdA, fumC, sdhC, mdh *were up-regulated (p < 0.01, p < 0.01, p < 0.01, p < 0.01, p < 0.01, respectively), and glyoxylate pathway gene *aceA *were up-regulated (p < 0.01) for *gdhA *mutant as compared to the wild type under N-rich condition. Note that *aspC *gene expression was down-regulated for the mutant as compared to the wild type (p < 0.05), which may be due to the coupling with GDH reaction. Moreover, Additional file 4f indicates that the respiratory chain genes such as *cydB, cyoA, ndh *were up-regulated (p < 0.01, p < 0.01, p < 0.1, respectively), and *sodA *gene expression was also up-regulated (p < 0.01), where the latter was consistent with the up-regulation of *soxR *gene expression (p < 0.05) (Additional file 4a) for *gdhA *mutant as compared to the wild type under N-rich condition.

**Figure 6:**
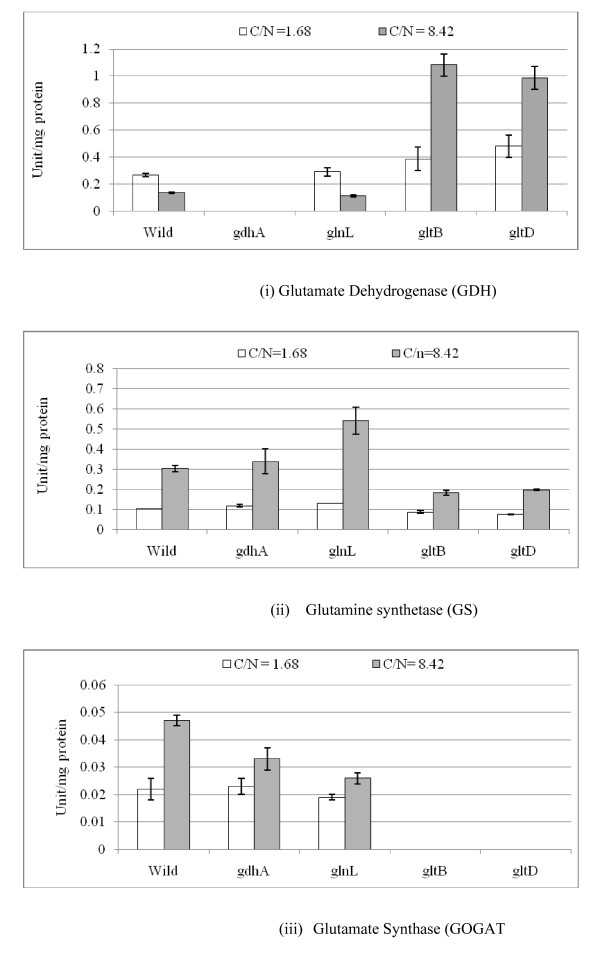
Enzyme Activities of N- assimilation pathway such as GDH, GS, and GOGAT in the wild type *E.coli *and its mutants in Units*/mg protein.

Under N-limitation, the transcript levels of *glnA, L*, *G *genes as well as *rpoN *were down-regulated (p < 0.01, p < 0.01, p < 0.01, p < 0.01, respectively), whereas transcript levels for *gltB, D *genes were similar to those of the wild type (Additional file 4d), indicating that only GOGAT pathway was active in *gdhA *mutant under N-limitation. It was also confirmed by the enzyme activity measurements (Additional file 4 and Fig. 6). Under N-limitation, the transcript level of *mlc *gene was higher (p < 0.01) (Additional file 4c) and correspondingly *ptsH *gene was down-regulated (p < 0.01) (Additional file 4g). Moreover, the transcript levels of TCA cycle genes such as *gltA, icdA, sdhC *were down-regulated (p < 0.05, p < 0.01, p < 0.01, respectively) for *gdhA *mutant as compared to the wild type (Additional file 4g).

### *glnL *mutant

The operon *glnALG *transcribes a sensor-histidine kinase (NtrB/NR_II_, *glnL*) which phosphorylates response regulator (NtrC/NR_I_, *glnG*) in response to the change in C/N ratio. This operon transcribes the only glutamine synthesizing enzyme, glutamine synthetase (GS, *glnA*), where the GS is an essential enzyme (*glnA *mutant failed to grow in M9 minimal medium, data not shown). In the *glnL *mutant under N- rich condition, the transcript level of *glnA *gene was comparable to that of the wild type (Additional file 5b), while it was significantly reduced (p < 0.01) under N- limitation (Additional file 5d). Additional file 5a shows that the transcript levels of genes, which encode global regulators, such as *fnr, arcA, cra, mlc, fur, soxR, S *and *rpoS *were up-regulated (p < 0.05, p < 0.01, p < 0.1, p < 0.01, p < 0.01, p < 0.01, p < 0.01 and p < 0.01 respectively) for *glnL *mutant as compared to the wild type under N- rich condition. Additional file 5e shows up-regulations of PTS and glycolysis genes such as *ptsH, ptsG, pfkA, pykF *(p < 0.01, p < 0.01, p < 0.01, p < 0.01, respectively), and PP pathway genes such as *zwf *and *gnd *genes (p < 0.01, p < 0.01, respectively) and ED pathway gene *eda *(p < 0.01) as compared to the wild type under N-rich condition. Moreover, Additional file 5e also indicates that the TCA cycle genes such as *gltA, icdA, sdhC, fumC, mdh *were up-regulated (p < 0.01, p < 0.01, p < 0.01, p < 0.01, p < 0.01, respectively) and glyoxylate pathway gene *aceA *as well as *lpdA *were up-regulated (p < 0.01, p < 0.01, respectively) for *glnL *mutant under N-rich condition. The up-regulations of TCA cycle genes are consistent with the increased specific CO_2 _production rate. The *aspC *gene expression was higher (p < 0.01) for *glnL *mutant as compared to the wild type under such condition (Additional file 5e).

Under N-limitation, the transcript level of *ptsH *gene was down-regulated (p < 0.1), while *yfiD *and *gadA *genes were up-regulated (p < 0.01, p < 0.1, respectively). Note that Additional file 5g indicates that the TCA cycle genes such as *gltA*, *icdA*, *sdhC*, *mdh *were down-regulated (p < 0.01, p < 0.01, p < 0.01, p < 0.01, respectively) and *lpdA *was down-regulated (p < 0.01) for *glnL *mutant as compared to the wild type under N-limitation. Additional file 5h, however, shows up-regulation of the transcript levels of respiratory pathway genes such as *cydB, cyoA*, and *ndh *(p < 0.01, p < 0.01, p < 0.01, respectively) for *glnL *mutant as compared to the wild type under N-limitation, which is consistent with the increased specific CO_2 _production rate.

### *gltB *and *gltD *mutants

The *gltBD *operon is responsible for the synthesis of glutamate synthase (GOGAT) enzyme. Under N- rich condition, GDH and GS may be used to form glutamate and glutamine. Additional file 6b shows significant down-regulation of the transcript level of *gdhA *gene (p < 0.01) for *gltB, D *mutants as compared to the wild type, where p-values are given for the critical values of either *gltB *or *gltD *mutant in the followings. Additional file 6a indicates that the transcript levels of *fur*, *rpoS *and *soxR/S *were up-regulated (p < 0.01, p < 0.01, p < 0.01, p < 0.01, respectively), while transcript expression of *mlc *gene was down-regulated (p < 0.01) for *gltB, D *mutants as compared to the wild type. The down-regulation of *mlc *transcript caused up-regulation of *ptsH *transcript (p < 0.01) as shown in Additional file 6e. The transcript of *glnE *gene which encodes ATase was down-regulated (p < 0.01) for *gltB, D *mutants as compared to the wild type under N-rich condition (Additional file 6b). Moreover, Additional file 6b shows that *rpoN*, *glnB, glnK, glnL*, and *glnD *genes were up-regulated (p < 0.01, p < 0.01, p < 0.01, p < 0.01, p < 0.01, respectively) for *gltB, D *mutants as compared to the wild type, implying the activation of GS pathway.

Under N-limitation, the transcript levels of such genes as *fur*, *rpoS *and *soxR *were up-regulated (p < 0.01, p < 0.01, p < 0.01, respectively), and also *mlc *and *cra *genes were up-regulated (p < 0.05, p < 0.01 respectively) for *gltB, D *mutants as compared to the wild type (Additional file 6c). Additional file 6g indicates that the transcript level of *ptsH *gene was down-regulated (p < 0.01), while the transcript levels of *pfkA *and *eda *genes were up-regulated (p < 0.01, p < 0.05) for *gltB, D *mutants as compared to the wild type under N-limitation. Moreover, the transcript levels of *lpdA, gltA*, and *sdhC *genes were down-regulated (p < 0.01, p < 0.01, p < 0.01, respectively), while *yfiD *and *gadA *genes were up-regulated (p < 0.01, p < 0.05, respectively) for *gltB, D *mutants as compared to the wild type under N-limitation. Additional file 6d indicates that the transcript levels of *glnA, glnG, glnK, nac*, and *glnE *genes were down-regulated (p < 0.01, p < 0.01, p < 0.01, p < 0.1, and p < 0.05, respectively) for *gltB, D *mutants as compared to the wild type, which implies that the cell tries to repress the GS activity. Note that *gdhA *gene expression was higher as compared to N- rich condition, and comparable to the wild type under N- limitation. The corresponding activity of GS showed repression, while that of GDH enzyme showed de-repression as compared to the wild type under N- limitation (Additional file 6 and Fig. 6).

## Discussion

The glucose concentration in the fermentor increased with the increase in C/N ratio (Fig. 2). The glucose uptake is made via phosphotransferase system (PTS) in *E.coli*, where phosphate of PEP is transferred by the phosphorelay via enzyme I (EI) encoded by *ptsI*, histidine phosphorylatable protein HPr encoded by *ptsH*, glucose specific enzyme II, EIIA^Glc ^encoded by *crr*, and membrane bound EIICB^Glc ^encoded by *ptsG*. When glucose is present in excess, the phosphorylated EIIA^Glc ^transfers phosphate to EIICB^Glc ^for the glucose uptake with phosphorylation, and the unphosphorylated EIIA^Glc ^is dominated in the cytosol [24]. Since unphosphorylated EIIA^Glc ^does not activate Cya, the cAMP level decreases under N-limitation together with *crp *gene as shown in Fig. 3a. Since *mlc *is under control of *crp*, the transcript level of *mlc *gene decreased as well (Fig. 3a), which caused up-regulation of transcript levels of *ptsH *and *ptsG *genes (Additional file 2). Moreover, increase in the glucose concentration at higher C/N ratio may have caused down-regulation of *cra*, which caused up-regulation of the glycolysis genes such as *ptsH*, *ptsG*, *pfkA*, *pykF*, together with *zwf *(Additional file 2).

The GDH pathway is important during glucose-limited (C-limited) condition. This pathway is favored when the organism is stressed for energy because GDH does not use ATP as does GS pathway [25]. Fig 3b shows the decreased expression of *gdhA *as C/N ratio increases. Liang and Houghton [26] investigated the effect of NH_4_Cl concentration on GDH and GS activities, and showed the up-regulations of GDH and transhydrogenase activities at lower NH_4_Cl concentration.

The availability of nitrogen is sensed by P_II _protein at the level of intracellular glutamine, where glutamine is synthesized by glutamine synthetase (GS) encoded by *glnA*, and is transported mainly by GlnHPQ. The *glnHPQ *operon is under the control of tandem promoters such as *glnHp1 *and *glnHp2*, where the former is σ^70 ^- dependent, and the latter is σ^54 ^- and NtrC-P dependent [27,28]. It has been shown that as the major transcriptional effector of the glucose effect, Crp affects nitrogen regulation [9]. Namely, *glnAp1 *is activated by Crp with glutamine as N-source. Through *glnHPQ*- dependent signaling, Crp acts to decrease the amount of the phosphorylated NtrC activator, which in turn causes the decrease in *glnAp2 *expression [9]. However, this regulation is more complex as explained next.

It has been suggested that σ^54^-dependent Ntr genes of *E.coli *form a gene cascade in response to N-limitation [29]. The central participants of Ntr response are NR_I _or NtrC and NR_II _or NtrB, and RNA polymerase complexed to σ^54^. NR_I _is the transcriptional activator of σ^54^-dependent promoters, while NR_II _is a bifunctional protein that can either transfer phosphate to NR_I _or control the dephosphorylation of NR_I _- phosphate. N-limitation results in the phosphorylation of NR_I_, which in turn stimulates the expression of *glnALG *operon. The expression of the *glnALG *operon is controlled by tandem promoters such as *glnAp1 *and *glnAp2*, where *glnAp1 *is a σ^70 ^- dependent weak promoter and its transcription can be activated by Crp and blocked by Ntr-P. On the other hand, *glnAp2 *is transcribed by RNA polymerase (Eσ^54^) and is activated by Ntr-P. Therefore, *glnAp2 *is responsible for activating *glnA *transcription under N-limitation [30]. Fig 3b shows that the expressions of *glnA, L, G *genes changed in similar fashion as *rpoN *gene expression.

It has been reported that there is no NR_I_-P binding sites in the *gdhA *regulatory region [31], and it is unlikely for NR_I _to directly repress *gdhA *promoter [32]. As it has been shown that Nac is involved in the transcriptional repression of *gdhA *gene under N-limitation [32], Nac seems to repress *gdhA *gene as shown in Fig. 2b. Fig 2b shows that the transcript level of *gdhA *gene was lower, while *gltB *and *D *genes were higher under N-limitation as compared to C-limitation. NADPH is an important cofactor in GDH and (GS)-GOGAT activities, and it has been reported that transhydrogenase plays some role in the regulation of these pathways [26]. Under N-limitation, the glutamate and glutamine synthetic pathways are expected to be repressed due to shortage of NH_3 _for those reactions, and thus NADPH is less utilized, resulting in overproduction of NADPH. Part of this may be converted to NADH by transhydrogenase and the converted NADH together with other NADH formed may be utilized for ATP production through respiratory chain. Overproduction of NADPH represses such pathways as G6PDH, 6PGDH and ICDH in *E.coli*. However, *zwf *was activated in Fig. 3c, which may be due to *soxR/S *caused by higher respiratory activity. The ICDH activity is reported to be insensitive to N concentration, where Fig. 3c also shows little change in *icdA *gene.

*E.coli *possesses two closely related P_II _paralogues such as GlnB and GlnK, where GlnB is produced constitutively, and it regulates the NtrB (NR_II_)/NtrC (NR_I_) two component system [33]. It has been shown that the intracellular concentrations of NR_I _and NR_II _increased upon N-limitation [34-36]. The phosphorylated NtrC is an activator of various nitrogen-controlled genes such as *glnA *which codes for GS [29] and *glnK *encoding the second P_II _paralogues [36]. The increased NR_I_, presumably in the phosphorylated form such as NR_I_-P activates the expression of *glnK *and *nac *promoters under N-limitation [37,38]. Fig 3b shows that the transcript levels of *glnK *and *nac *gene increased as C/N ratio increases, while slight decrease can be seen at the highest C/N ratio, where it has been reported that *glnK *and *nac *promoters are sharply activated when ammonia is used up [36].

The *gltBDF *operon which has been found to have binding affinity with global regulators such as Fnr and Crp in the promoter region [39], where the transcript level of *fnr *gene was higher under N-limitation whereas *crp *gene became lower (Fig. 3a). The up-regulation of *yfiD *(Fig. 3c) may be due to up-regulation of *fnr*.

**Figure 7:**
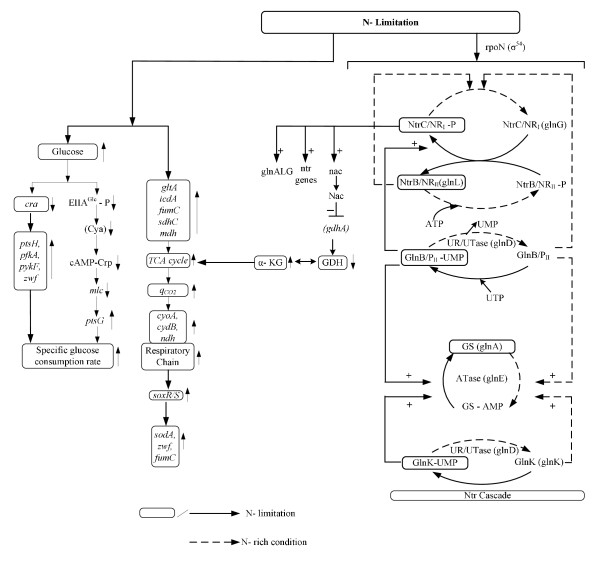
**Proposed overall mechanism of N- assimilation in *E.coli *under C- limited (N- rich) and N- limited conditions**.

The Ntr system is composed of four enzymes (Fig. 7): a uridylytransferase/uridylyl-removing enzyme (UTase/UR) encoded by *glnD *gene, a small trimeric protein, P_II _encoded by *glnB*, and the two-component system composed of NtrB and NtrC. GlnD controls the activity of GS by adenylylation/deadenylylation through a bifunctional enzyme adenylyltransferase (ATase), the *glnE *gene product [40-42]. The activity of GlnK becomes high under N-limitation (Fig. 3b) and contributes to the regulation of NtrC-dependent genes [43]. It has been shown that on GS adenylylation, ATase activity is regulated by UTase/UR and P_II _such that upon nitrogen limitation, UTase covalently modifies P_II _by addition of a UMP group at a specific residue and the resultant uridylylated form of P_II _promotes deadenylylation of GS by ATase (Fig. 7). Conversely, under N- rich condition, the uridylyl-removing activity of GlnD predominates and the deuridylylated P_II _promotes adenylation of GS by ATase. Adenylylation by ATase is promoted by deuridylated P_II _which is produced by UR action on P_II _(UMP)_3 _under higher N-concentration (low C/N ratio) (Fig. 7). These indicate that UTase/UR and P_II _acting together sense the intracellular nitrogen status [44]. The P_II _signal transduction proteins such as GlnB and GlnK are uridylylated/deuridylylated in response to intracellular glutamine level, where low intracellular glutamine level, signalling N-limitation, leads to uridylylation of GlnB [44]. GlnB was shown to be allosterically regulated by α-KG, and thus GlnB may play a role in integrating signals of C/N status. The NtrB/NtrC two component system and GlnE which adenylylates/deadenylylates GS are the receptors of GlnB signal transduction [43]. It has been suggested that the carbon/cAMP effect was mediated through GlnB uridylylation [43].

The phosphorylated NR_I_/NtrC (NR_I_/NtrC-P) activates transcription from N- regulated σ^54^-dependent promoters by binding to the enhancers [11,44-46]. P_II _and the related GlnK protein control the phosphorylation state of NR_II_/NtrB by stimulating the phosphatase activity of NR_II _(Fig. 7). The ability of GlnK and P_II _to regulate the activities of NR_II _is in turn regulated by the intracellular signals of C and N availability via allosteric control [11].

It is not clear at this time why TCA cycle is activated under N- limitation. One of the possible reasons why TCA cycle together with respiration became active under N- limitation may be due to the accumulation of α-KG caused by the blockage of the GDH pathway. This is also true for *gdhA *mutant even under N- rich condition. The reason why glycolysis was activated may be due to ATP requirement and the down-regulations of *crp *and *cra *caused by the increase in glucose concentration as mentioned before. When *gdhA *gene was knocked out, the changing patterns of nitrogen-regulated genes under N-rich condition (Additional file 4b) were similar to those of the wild type under N-limiting condition (Fig. 3b), indicating that GS-GOGAT cycle was mainly utilized, where 2 moles of Glu were formed from α-KG and Gln via GOGAT pathway, and Gln is formed from Glu via GS pathway. Under N-limitation, GS became less active for *gdhA *mutant, where only GOGAT pathway was active (Additional file 4d).

**Figure 8:**
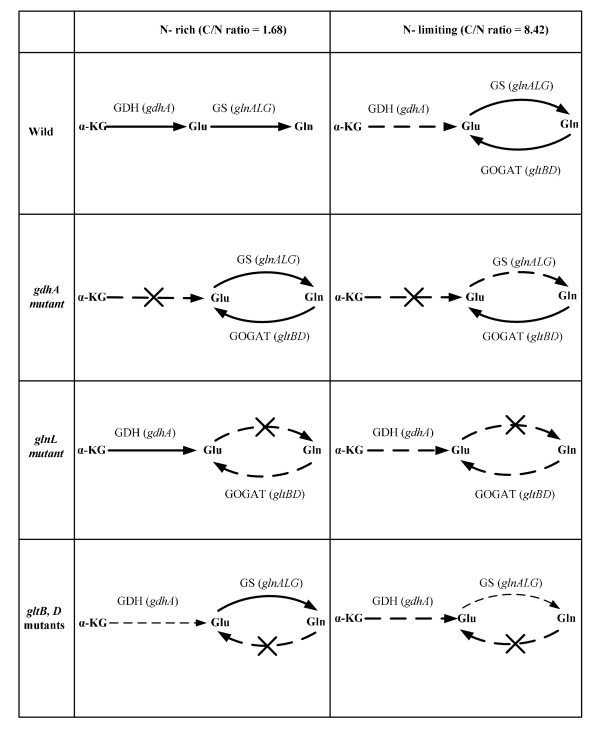
**Proposed schematic illustration of N-assimilation pathways for the wild type *E.coli *and its mutants under C- limiting (N-rich) and N-limiting conditions**.

**Figure 9:**
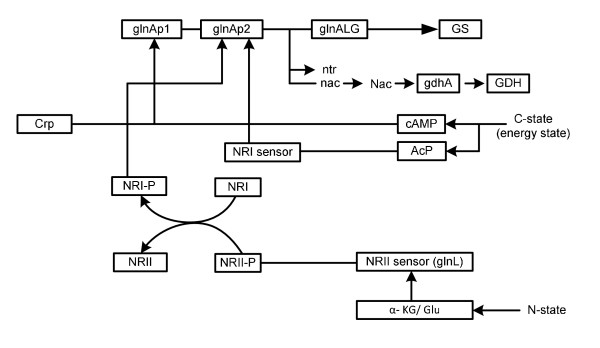
**Proposed scheme of N- assimilation in *E.coli *in response to the changes in C- and N-concentrations**.

In the case of *glnL *mutant, the transcripts of glycolysis, PP pathway, and TCA cycle genes were up-regulated under N-rich condition, while those were repressed under N-limitation. Part of the reason may be due to up-regulation of *crp *and *mlc *genes. Under N-limitation, Glu is produced from both GDH and GOGAT but the former is less utilized because of less availability of NH_3_, which means that essentially GOGAT pathway was active. This phenomenon is similar to the case of *gdhA *mutant under N-limitation (Fig. 8). Since NR_I _could not be phosphorylated in *glnL *mutant, *glnA *gene expression was lower as compared to the wild type (Additional file 5d). Moreover, the transcript level of *nac *gene did not change even under N- limitation, which may be due to lack of phosphorylated NR_I_. There might be another mechanism in *glnL *mutant [47]. Namely, acetyl phosphate (AcP) is a signaling molecule for glucose availability [48] as well as cAMP, and NR_I _itself is capable of sensing the AcP level, where this becomes significant only in the absence of NR_II _(*glnL *mutant) [49,50]. In the absence of NR_II_, NR_I _senses AcP level and induce *glnAp2*. The *glnG *gene expression may be reflected in the carbon level, where NR_I _binding site overlap another promoter, *glnAp1*, which is regulated by cAMP-Crp. The effect of *glnG *gene knockout is also given elsewhere [51]. Those mechanisms are briefly summarized in Fig. 9.

In the case of *gltB, D *mutants, the effects of these genes knockout on the metabolism may be considered to be minor under N-rich condition, since this pathway is not utilized under N-rich condition in the wild type. However, it was shown that *gdhA *gene expression was significantly reduced, while GS pathway was activated as can be seen in Additional file 6b. Since, the transcript level of *rpoN *gene was high even under N-rich condition for *gltB, D *mutants (Additional file 6b), this may have caused Nac to be increased (although not significant in Additional file 6b) and repressed *gdhA *gene. It is not clear at this time why the transcript levels of *rpoN *gene became higher under N-rich condition. Under N- limitation, *nac *gene was repressed (p < 0.01), and thus the transcript level of *gdhA *gene was comparable to the wild type under N-limitation. The de-repression of GDH activity appears to be driven by the cellular requirement for glutamate (Additional file 6 and Fig. 6). Additional file 6d shows that the transcript levels of *glnA *and *glnG *genes were low (p < 0.01, p < 0.01) and thus GS enzyme was repressed under N- limitation as compared to the wild type (Additional file 6 and Fig. 6). Additional file 6e and 6g indicates that the transcript level of *gadA *gene was higher under both C-limitation and N-limitation. Since *gltB, D *mutants are reported to be osmosensitive [52], glutamate and GABA etc. may have been accumulated and excreted into the fermentation broth. Overall, the de-repression of GDH enzyme under N- limitation indicates the complex mechanism of N- regulation in these mutants and the mechanistic details of this de-repression are not yet completely known.

One of the reason why the cell concentrations of the mutants under C- limitation is lower than that of the wild type (Fig. 4a) may be due to lower formation rate of Glu caused by the deficiency in N- assimilating pathways. The overall regulation mechanism is summarized in Fig. 7, and the effects of specific gene knockout on the nitrogen assimilating pathways are illustrated in Fig. 8.

It is important to note that the experimental results at C/N ratio 16.48 show somewhat different pattern as compared to the other ratios used in the present study. The amount of available nitrogen seems to be the crucial factor in deciding the metabolic response especially under N- limitation. Most the pathways used for N- assimilation under N- limitation utilize high amount of ATP. Therefore, it appears critical for the cell to shut down activities of such pathways like GS-(GOGAT) under certain circumstances to save ATP and to prevent excessive glutamine production. This scenario has been speculated for ammonium shock to the carbon starved cells [20]. Note that the transcript levels of such genes as *glnA, glnL, glnG *and *gltB, gltD *which encodes for GS-GOGAT pathway enzymes were reduced (Fig. 3b).

## Conclusion

The metabolic regulation of *E.coli *was clarified to some extent under both C- limitation (N- rich condition) and N- limitation in view of fermentation characteristics, transcript levels, and enzyme activities. The overall mechanism was found to be as depicted in Fig. 7. Moreover, the effects of knockout of N- assimilation pathway genes such as *gdhA, glnL*, and *gltB, D *were investigated and found to be as shown in Fig. 8.

## Materials and methods

### Strains, media composition, and cultivation conditions

The strains used were *E.coli *BW25113 [F^- ^λ^- ^*rph-1 *Δ*araBADAH33 lacI*^*q*^Δ*lac*Z_WJ16 _*rrn*B_T14 _Δ*rhaBAD*_LD78 _*hsd*R514], and its single gene knockout mutants lacking such genes as *gltB *(JW3180), *gltD *(JW3180), *gdhA *(JW1750), and *glnL *(JW3840). These single gene knockout mutants were obtained from Keio collection [53]. All the strains were first precultured in the Luria-Bertani medium. The second preculture and the main culture were carried out using M9 minimal medium containing 10 g of glucose together with the following components (per liter): 6.81 g Na_2_HPO_4_, 2.99 g KH_2_PO_4, _0.58 g NaCl and 5.94 g (NH_4_)_2_SO_4_. The following components were filter sterilized and then added (per liter) with 1 ml of 1 M MgSO_4_.7H_2_O, 1 ml of 0.1 mM CaCl_2_.2H_2_O, 1 ml of 1 mg/l thiamine HCl and 10 ml of trace element solution containing (per liter): 0.55 g CaCl_2_.2H_2_O, 1.67 g FeCl_3_.6H_2_O, 0.1 g MnCl_2_.4H_2_O, 0.17 g ZnCl_2_, 0.043 g CuCl_2_.2H_2_O, 0.06 g CoCl_2_.2H_2_O, and 0.06 g Na_2_MoO_4_.2H_2_O. The nitrogen concentrations used in the present experiments were 0.594 g/l, 1.188 g/l, 2.376 g/l, 3.564 g/l, and 5.94 g/l of (NH_4_)_2_SO_4_, whereas the concentrations of all the other medium components were the same. The continuous culture was conducted in a 1-l fermenter (MDL 100, Marubishi Co., Tokyo, Japan) with a working volume of 500 ml. The pH was controlled at 7.0 ± 0.05 using 2 N HCl or 2 N NaOH, and the temperature was set at 37°C. The air flow rate was 1 vvm (air volume/working volume/min), and the agitation speed was 350 rpm to maintain the dissolved oxygen concentration to be at 35-40% (v/v) of air saturation [22]. The CO_2 _and O_2 _concentrations were monitored using an off-gas analyzer (BMJ-02 PI, ABLE Co., Japan). The dilution rate was 0.2 h^-1 ^for all the continuous cultures. The samples were collected at the steady state which was confirmed by the constant off-gas and cell density. It generally took 5-6 residence times to achieve the steady state.

### Analytical method

Bacterial growth was monitored by measuring the optical density of the culture broth at 600 nm (OD_600 nm_) using a spectrophotometer (Ubet-30, Jasco, Tokyo, Japan). It was converted to dry cell weight (DCW) based on the OD_600 nm_-DCW relationship previously obtained [54]. Glucose and acetate concentrations in the medium were measured using commercially available kits (Wako Co., Osaka, Japan for glucose; Roche, Molecular Biochemical, Mannheim, Germany for acetate).

### RNA preparation, design of PCR primers

Total RNA was isolated from *E. coli *cells by Qiagen RNeasy Mini Kit (QIAGEN K.K., Japan) according to the manufacturer's recommendation. The quantity and purity of RNA were determined by the optical density measurements at 260 and 280 nm and by 1% formaldehyde agarose gel electrophoresis. The sequences of primers for respective genes used in this study were reported elsewhere [55], except such genes as *rpoN, glnA, glnB, glnD, glnE, glnG, glnL, gltD *and *nac*. The primer sequences of these additional genes are as follows:

*rpoN*

Forward: 5' GCAACTCAGGCTTAGCCAAC 3'

Reverse: 5' TCCAGCGTTTCACTGTCTTG 3'

*glnA*

Forward: 5' ATGTCCGCTGAACACGTACT 3'

Reverse: 5' GCTGTAGTACAGCTCAAACTC 3

*glnB*

Forward: 5' CGAAGTGAAAGGCTTTGGTC 3'

Reverse: 5' GCCACGTCAAAGACGAAGAT 3'

*glnD*

Forward: 5' CACCTGTTGATGTCGGTGAC 3'

Reverse: 5' GCTTCCAGCTATTCCACAGC 3'

*glnE*

Forward: 5' CCCGCACCACCTATTTAGAA 3'

Reverse: 5' GCTGGTAAAGGGTGTTTGGA 3'

*gln G*

Forward: 5' ATGCAACGAGGGATAGTCTG3'

Reverse: 5' TCACTCCATCCCCAGCTCTT 3'

*glnL*

Forward: 5' GAGATGGCTCCGATGGATAA3'

Reverse: 5' ATGGGTCAGGTAACGCTTTG 3'

*gltD*

Forward: 5' CAATTTATCGACCTGCAGCG 3'

Reverse: 5' AACTTCCAGCCAGTTCATAAT 3'

*nac*

Forward: 5'TTCAGACGCCTGAAATACTTC3'

Reverse: 5' TTAGCTCACCAATTGCCACT 3'

Criteria for the design of the gene-specific primer pairs were followed according to Sambrook and Russell [56]. The primers used in this study were synthesized at Hokkaido System Science Co. (Sapporo, Hokkaido, Japan). In all cases, the primer-supplied company confirmed the purity and absolute specificity of primers.

### c DNA synthesis and PCR amplification

RT-PCR reactions were carried out in a TaKaRa PCR Thermal Cycler (TaKaRa TP240, Japan) using Qiagen OneStep RT-PCR Kit (QIAGEN K.K., Japan). The reaction mixture was incubated for 30 min at 50°C for reverse transcription (cDNA synthesis) followed by 15 min incubation at 95°C for initial PCR activation. Then, the process was subjected to 30 cycles of amplification which consisted of a denaturing step (94°C for 1 min), an annealing step (approximately 5°C below melting temperature of primers for 1 min) and an extension step (72°C for 1 min), and finally the reaction mixture was incubated for 10 min at 72°C for final extension. To check for nucleic acid contamination, one negative control was run in every round of RT-PCR. This control lacks the template RNA in order to detect possible contamination of the reaction components. 5 ml of amplified products were run on a 1% agarose gel. Gels were stained with 1 μg ml^-1 ^of ethidium bromide, photographed using a Digital Image Stocker (DS-30, FAS III, Toyobo, Osaka, Japan) under UV light and analyzed using Gel-Pro Analyzer 3.1 (Toyobo, Osaka, Japan) software. Although the PCR products obtained for all the genes showed the predicted sizes on agarose gel, the identity of amplified fragments of some genes was demonstrated by DNA sequencing. In order to determine the optimal amount of input RNA, the two-fold diluted template RNA was amplified in RT-PCR assays under identical reaction conditions to construct a standard curve for each gene product. When the optimal amount of input RNA was determined for each gene product, RT-PCR was carried out under identical reaction conditions to detect differential transcript levels of genes. The gene *dnaA*, which encodes for DnaA, a replication initiation factor in *E.coli *and is not subjected to variable expression, i.e. abundant expression at relatively constant rate in most cells, was used as an internal control for the RT-PCR determinations [55]. To calculate the standard deviation, RT-PCR was independently performed three times for each gene under identical reaction condition. To ensure that the observed changes were statistically significant, the Student's t-test was applied.

### Enzyme Assays

The cells were harvested at the same stage as those taken for RT-PCR analysis by the centrifugation at 10,000 × *g *for 10 min, washed twice in ice-cold 100 mM Tris-HCl (pH 7.0) buffer containing 20 mM KCl, 5 mM MnSO_4_, 2 mM dithiothreitol and 0.1 mM EDTA, and then re-suspended in the same buffer solution (ca. 15 g wet cells in 50 ml buffer solution) and stored at -80°C in aliquots for at least 30 min. The cell disruption was achieved by sonication on an ultrasonic disrupter (UD-201, Tomy Co., Tokyo, Japan) and resulting crude cell extracts were immediately used for the measurements of enzyme activities or stored at -80°C in aliquots. All above mentioned operations were carried out on ice [54]. In the present study enzyme activities involved in the N- assimilation pathway were measured. The measurements were carried out on a thermostat recording spectrophotometer (U-2000A, Hitachi Co., Japan) at 37°C. The protein concentrations were estimated by the Bradford assay method. Each enzyme was measured three times for the same culture. GDH was assayed by following the oxidation of NADPH in a solution containing 50 mM Hepes/KOH, pH 7.5, 50 mM NH_4_Cl, 5 mM α- KG, and 0.3 mM NADPH. GOGAT was assayed in the same reaction mixture substituting 5 mM L-glutamine for NH_4_Cl [57]. GS assay followed the method suggested by Sigma-Aldrich based on previously published study [58].

## Abbreviations

PEP: Phospho-enol-pyruvate; PYR: Pyruvate; OAA: Oxaloacetic Acid; α-KG: 2-ketoglutarate; PTS: Phosphotransferase system; GS: Glutamine Synthetase; GOGAT: Glutamate synthase; GDH: Glutamate dehydrogenase; PHB: poly (3-hydroxybutyrate); GABA: Gamma-aminobutyric acid; DCW: Dry Cell Weight.

## Competing interests

The authors declare that they have no competing interests.

## Authors' contributions

RK carried out fermentation experiments, assayed, made statistical analysis, analyzed the result, and drafted the manuscript. KS participated in the experimental design, analyzed the result, and prepared manuscript together with RK. All authors read and approved the final manuscript.

## Supplementary Material

Additional file 1 Fermentation parameters for the chemostat cultures of the wild type *E.coli *at the dilution rate of 0.2 h^-1 ^under various C/N ratios. Click here for file

Additional file 2 Global regulators and their regulated genes. Click here for file

Additional file 3 **a**: **Fermentation parameters for the chemostat cultures of the wild type ***E.coli ***in comparison to the nitrogen regulatory mutants at the dilution rate of 0.2 h^-1 ^at 100% nitrogen.b**: Fermentation parameters for the chemostat cultures of the wild type *E.coli *in comparison to the nitrogen regulatory mutants at the dilution rate of 0.2 h^-1 ^at 20% nitrogen concentration. Click here for file

Additional file 4 Comparison of the transcriptional mRNA levels between the wild type *E.coli *and *gdhA *mutant genes at C/N ratio 1.68 and 8.42. Click here for file

Additional file 5 Comparison of the transcriptional mRNA levels between the wild type *E.coli *and *glnL *mutant genes at C/N ratio 1.68 and 8.42. Click here for file

Additional file 6 Comparison of the transcriptional mRNA levels between the wild type *E.coli *and *gltB, gltD *mutants genes at C/N ratio 1.68 and 8.42. Click here for file
