# Supplementary material for: Metabolic regulation of Escherichia coli and its gdhA, glnL, gltB, D mutants under different carbon and nitrogen limitations in the continuous culture
Source: Microb Cell Fact. 2010 Jan 27;9:8. doi: 10.1186/1475-2859-9-8 (PMC2827463; doi:10.1186/1475-2859-9-8)
Supplement: Additional file 1 — Fermentation parameters for the chemostat cultures of the wild type E.coli at the dilution rate of 0.2 h-1 under various C/N ratios. [file 1475-2859-9-8-S1.DOC]

**Additional file 1**: Fermentation parameters for the chemostat cultures of the wild type *E.coli*

at the dilution rate of 0.2 h^-1^ under various C/N ratios

| Medium Components* ↓ |  | | | | |
| --- | --- | --- | --- | --- | --- |
| Glucose (g/l) 🡪 | 10 | | | | |
| Ammonium Sulfate (g/l) 🡪 | 5.94 | 3.56 | 2.38 | 1.19 | 0.59 |
| C/N ratio (percent w.r.t highest ammonium sulfate concentration) 🡪 | 1.68 (100) | 2.81 (60) | 4.21 (40) | 8.42 (20) | 16.84 (10) |
| Fermentation parameters ↓ |  |  |  |  |  |
| Biomass (g/l) | 3.33±0.17 | 3.21±0.16 | 2.90±0.15 | 1.67±0.08 | 0.82±0.04 |
| Glucose (g/l) | ND** | 0.04±0.00 | 0.12±0.01 | 3.03±0.15 | 5.93±0.30 |
| Acetate (g/l) | 1.73±0.09 | 1.52±0.08 | 1.43±0.07 | 1.41±0.07 | 0.96±0.05 |
| Cell yield (g/g) | 0.33±0.02 | 0.32±0.02 | 0.29±0.01 | 0.24±0.01 | 0.20±0.01 |
| Specific glucose consumption rate  (mmol/gdcw.h) | 3.33±0.17 | 3.44±0.17 | 3.78±0.19 | 4.65±0.23 | 5.51±0.28 |
| Specific acetate production rate  (mmol/gdcw.h) | 1.73±0.09 | 1.57±0.08 | 1.64±0.08 | 2.82±0.14 | 3.91±0.20 |
| Specific CO_2_ production rate  (mmol/gdcw.h) | 6.74±0.34 | 7.82±0.39 | 8.07±0.40 | 8.25±0.41 | 8.5±0.43 |

*Only carbon and nitrogen mentioned here. For detailed medium composition refer to materials and methods section

**ND = Not Detected
