# Supplementary material for: Metabolic regulation of Escherichia coli and its gdhA, glnL, gltB, D mutants under different carbon and nitrogen limitations in the continuous culture
Source: Microb Cell Fact. 2010 Jan 27;9:8. doi: 10.1186/1475-2859-9-8 (PMC2827463; doi:10.1186/1475-2859-9-8)
Supplement: Additional file 2 — Global regulators and their regulated genes. [file 1475-2859-9-8-S2.DOC]

**Additional file 2**: Global regulators and their regulated genes

| Global Regulators | Regulation | Regulated Genes |
| --- | --- | --- |
| Cra | *+* | *aceA, acnA, fbp, icdA, pckA, ppsA* |
|  | *-* | *acnB, eda, edd, eno, gapA, pfkA, pykF, ptsHI* |
| arcA/B | *+* | *pfl, cyd* |
|  | *-* | *aceBAK, aceEF, acnA, fumAC, gltA, icdA, lpdA, mdh, ptsG, cyo, sdhCDAB* |
| Fnr | *+* | *frd, pfl* |
|  | *-* | *acnA, fumAC, icdA, lpdA, ptsG, sdhCDAB, talA* |
| Mlc | *+* | *ldhA* |
|  | *-* | *crr, ptsG, ptsHI, manXYZ, malT* |
| Crp/Cya | *+* | *mlc, aceEF, acnAB, crr, fumA, gltA, mdh, pckA, ptsG, ptsHI, sdhABCD, sucABCD, tpiA, ompF* |
|  | *-* | *aceAB, lpd* |
| SoxR/S | + | *sodA*, *zwf, rpoD, rpoS, fumC, tolC, micF, marA* |
|  | - | *rob* |
| RpoS | + | *gadA, gadB, osmB, sodC, talA, talB* |
|  | - | *ompF, fnr, yfiD, ansB,* |
| Fur | + | *sodB, aceAB, ompF, sdhABCD, sucABCD, fumA* |
|  | - | *aceEF, ackA, ptsG, yncE, fecABE* |
