# Supplementary material for: Metabolic regulation of Escherichia coli and its gdhA, glnL, gltB, D mutants under different carbon and nitrogen limitations in the continuous culture
Source: Microb Cell Fact. 2010 Jan 27;9:8. doi: 10.1186/1475-2859-9-8 (PMC2827463; doi:10.1186/1475-2859-9-8)
Supplement: Additional file 3 — a: Fermentation parameters for the chemostat cultures of the wild type E.coli in comparison to the nitrogen regulatory mutants at the dilution rate of 0.2 h-1 at 100% nitrogen.b: Fermentation parameters for the chemostat cultures of the wild type E.coli in comparison to the nitrogen regulatory mutants at the dilution rate of 0.2 h-1 at 20% nitrogen concentration. [file 1475-2859-9-8-S3.DOC]

**Additional file 3**

**a**: Fermentation parameters for the chemostat cultures of the wild type *E.coli* in comparison to the nitrogen regulatory mutants at the dilution rate of 0.2 h^-1^ at 100% nitrogen concentration

| Medium Components* ↓ |  | | | | |
| --- | --- | --- | --- | --- | --- |
| Glucose (g/l) 🡪 | 10 | | | | |
| Ammonium Sulfate (g/l) 🡪 | 5.94 | | | | |
| C/N ratio (percent w.r.t highest ammonium sulfate concentration) 🡪 | 1.68 (100) | | | | |
| Fermentation parameters ↓ | Wild | *gdhA* | *glnL* | *gltB* | *gltD* |
| Biomass (g/l) | 3.33±0.17 | 2.82±0.14 | 2.66±0.13 | 2.70±0.14 | 2.52±0.13 |
| Glucose (g/l) | ND** | ND** | ND** | ND** | ND** |
| Acetate (g/l) | 1.73±0.09 | 2.21±0.11 | 1.82±0.09 | 2.49±0.12 | 2.43±0.12 |
| Cell yield (g/g) | 0.33±0.02 | 0.28±0.01 | 0.27±0.01 | 0.27±0.01 | 0.25±0.01 |
| Specific glucose consumption rate  (mmol/gdcw.h) | 3.33±0.17 | 3.94±0.20 | 4.17±0.21 | 4.11±0.21 | 4.40±0.22 |
| Specific acetate production rate  (mmol/gdcw.h) | 1.73±0.09 | 2.58±0.13 | 2.28±0.11 | 3.07±0.15 | 3.21±0.16 |
| Specific CO_2_ production rate  (mmol/gdcw.h) | 6.74±0.34 | 6.84±0.34 | 8.16±0.41 | 8.31±0.42 | 8.32±0.42 |

*Only carbon and nitrogen mentioned here. For detailed medium composition refer to materials and methods section **ND = Not Detected

**b:** Fermentation parameters for the chemostat cultures of the wild type *E.coli* in comparison to the nitrogen regulatory mutants at the dilution rate of 0.2 h^-1^ at 20% nitrogen concentration

| Medium Components*↓ |  | | | | |
| --- | --- | --- | --- | --- | --- |
| Glucose (g/l) 🡪 | 10 | | | | |
| Ammonium Sulfate (g/l) 🡪 | 1.19 | | | | |
| C/N ratio (percent w.r.t highest ammonium sulfate concentration) 🡪 | 8.42 (20) | | | | |
| Fermentation parameters ↓ | Wild | *gdhA* | *glnL* | *gltB* | *gltD* |
| Biomass (g/l) | 1.67±0.08 | 1.62±0.08 | 1.61±0.08 | 1.69±0.08 | 1.62±0.08 |
| Glucose (g/l) | 3.03±0.15 | 3.52±0.18 | 3.21±0.16 | 3.15±0.16 | 3.31±0.17 |
| Acetate (g/l) | 1.41±0.07 | 1.45±0.07 | 1.30±0.07 | 1.39±0.07 | 1.37±0.07 |
| Cell yield (g/g) | 0.24±0.01 | 0.25±0.01 | 0.24±0.01 | 0.25±0.01 | 0.24±0.01 |
| Specific glucose consumption rate  (mmol/gdcw.h) | 4.65±0.23 | 4.43±0.22 | 4.68±0.23 | 4.49±0.22 | 4.59±0.23 |
| Specific acetate production rate  (mmol/gdcw.h) | 2.82±0.14 | 2.98±0.15 | 2.68±0.13 | 2.74±0.14 | 2.82±0.14 |
| Specific CO_2_ production rate  (mmol/gdcw.h) | 8.25±0.41 | 7.27±0.36 | 7.32±0.37 | 7.54±0.38 | 7.59±0.38 |

*Only carbon and nitrogen mentioned here. For detailed medium composition refer to materials and methods section
