# Supplementary material for: Metabolic regulation of Escherichia coli and its gdhA, glnL, gltB, D mutants under different carbon and nitrogen limitations in the continuous culture
Source: Microb Cell Fact. 2010 Jan 27;9:8. doi: 10.1186/1475-2859-9-8 (PMC2827463; doi:10.1186/1475-2859-9-8)

**Additional file 6** Comparison of the transcriptional mRNA levels between the wild type *E.coli* and *gltB, gltD* mutants genes at C/N ratio 1.68 and 8.42


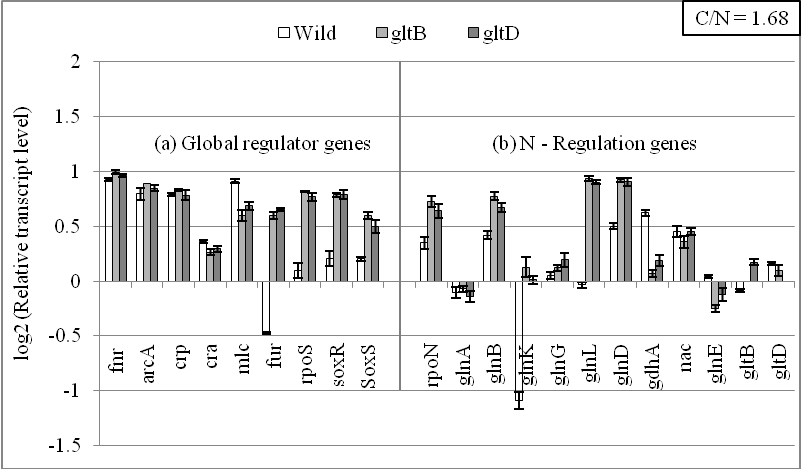


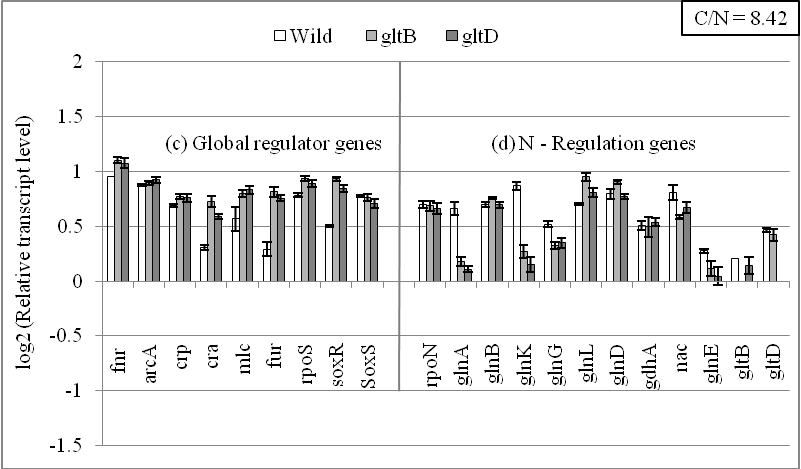


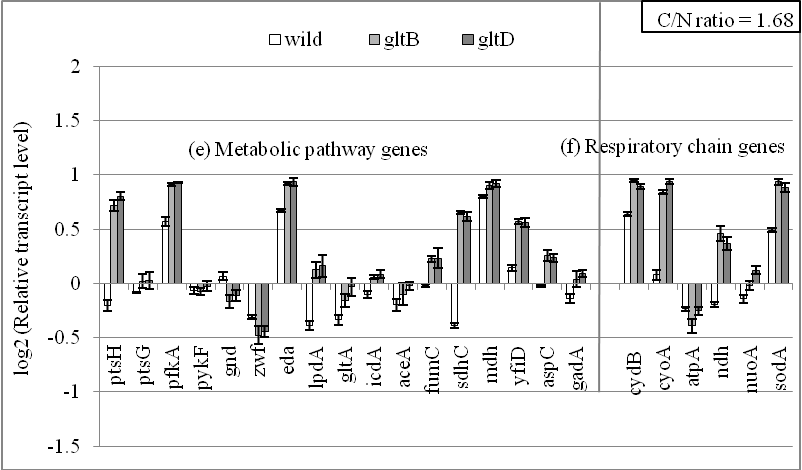


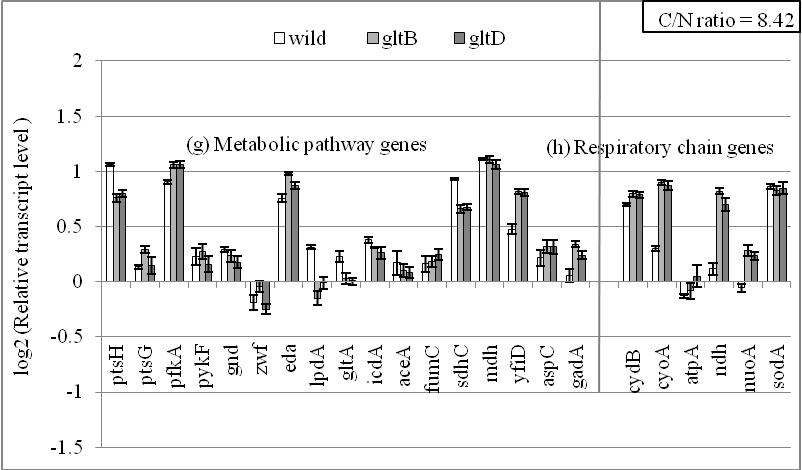

Supplement: Additional file 6 — Comparison of the transcriptional mRNA levels between the wild type E.coli and gltB, gltD mutants genes at C/N ratio 1.68 and 8.42. [file 1475-2859-9-8-S6.DOC]
